# Supplementary material for: The structural basis of proton driven zinc transport by ZntB
Source: Nat Commun. 2017 Nov 3;8:1313. doi: 10.1038/s41467-017-01483-7 (PMC5670123; doi:10.1038/s41467-017-01483-7)
Supplement: Supplementary file 1 — Supplementary Information [file 41467_2017_1483_MOESM1_ESM.pdf]

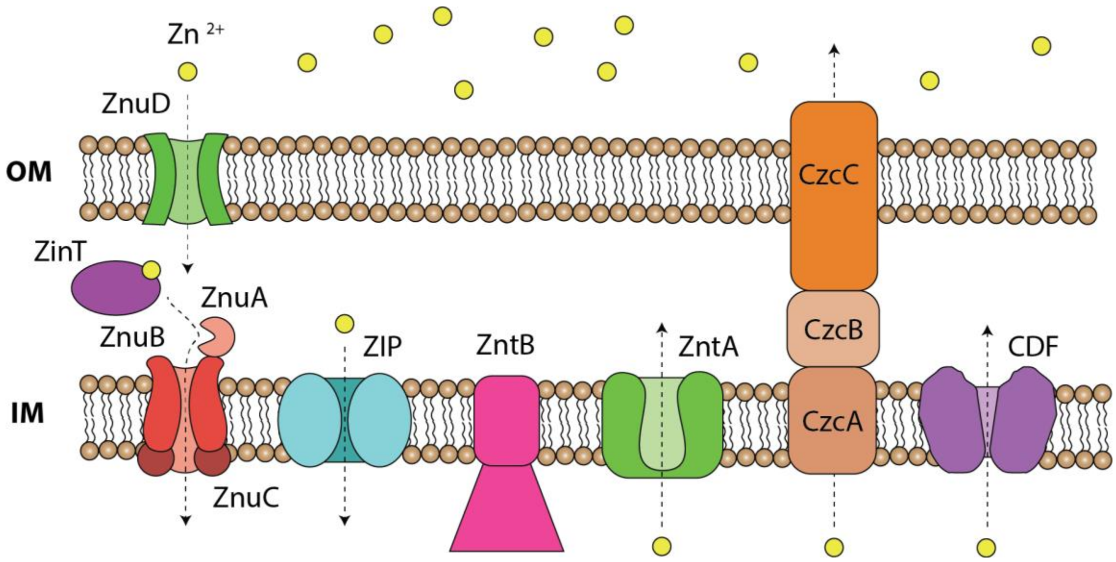


**Supplementary Figure 1. Schematic overview of membrane transporters involved in $Zn^{2+}$ homeostasis in Gram-negative bacteria.** $Zn^{2+}$ shown as yellow spheres, arrows pointing down and up show import and export respectively. OM and IM indicate outer and inner membranes respectively.

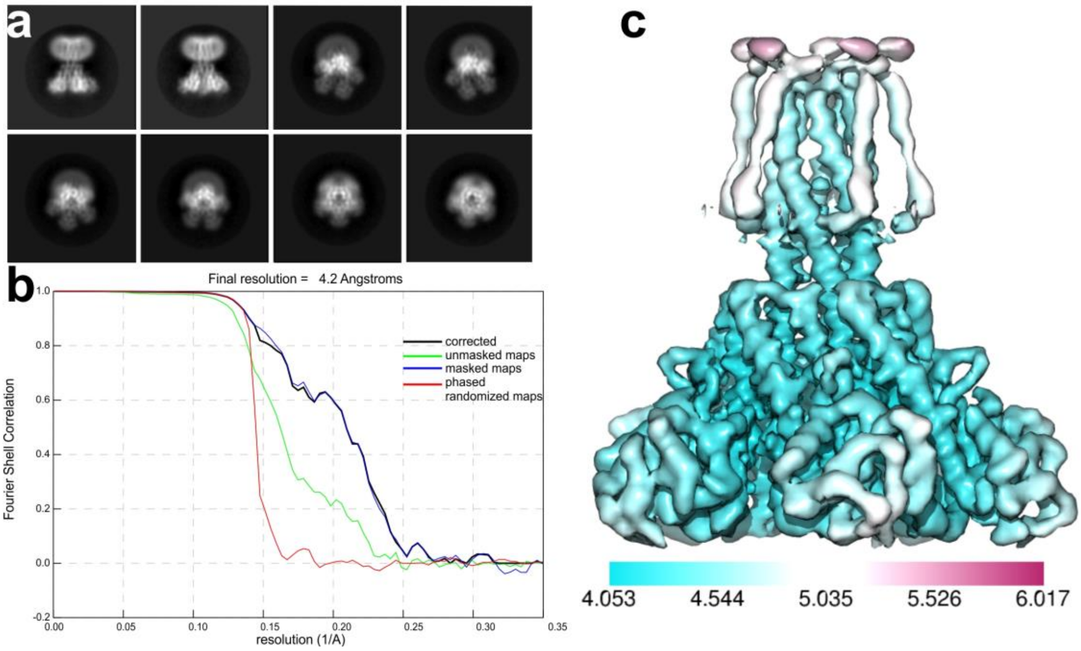


**Supplementary Figure 2 Cryo-EM reconstruction. (a)** Representative reference-free 2D class averages **(b)** Resolution estimation of the EM structure **(c)** Colour-coded resolution variations

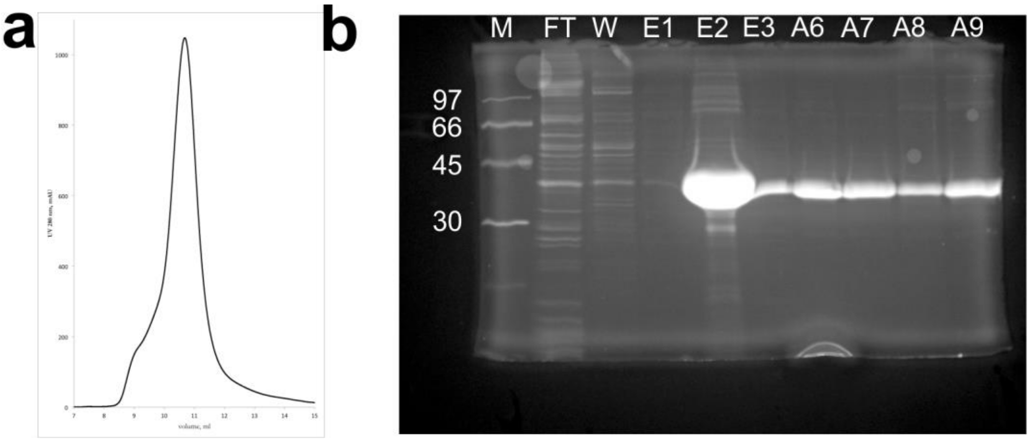


**Supplementary Figure 3 ZntB purification. (a)** Size-exclusion chromatography profile **(b)** SDS-PAGE gel, where M is a marker, FT – flow through, W – wash, E1-E3 – elution fractions and A6-A9 collected fractions.

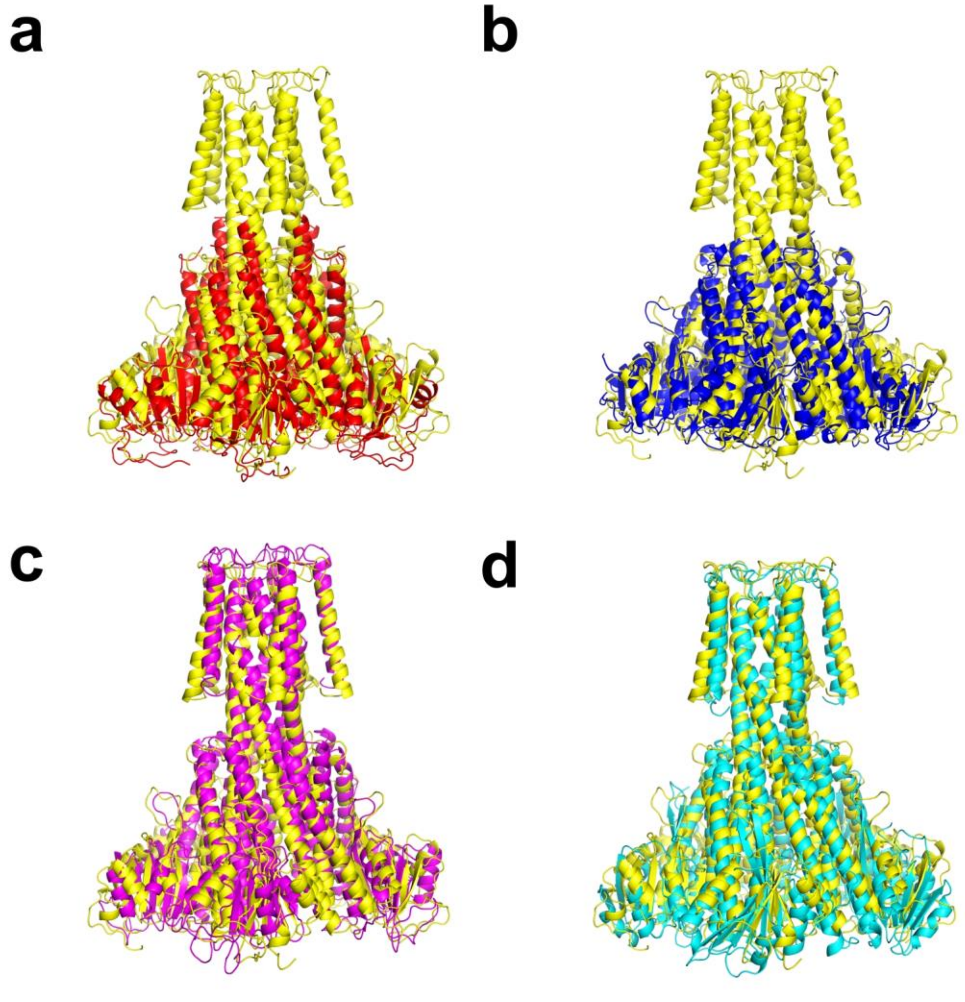


**Supplementary Figure 4 Structural comparison with (RMSD values in Å) (a)** soluble domain of StZntB (~12) **(b)** soluble domain of VpZntB (~2.5) **(c)** MjCorA (~5.8) **(d)** TmCorA (~12)

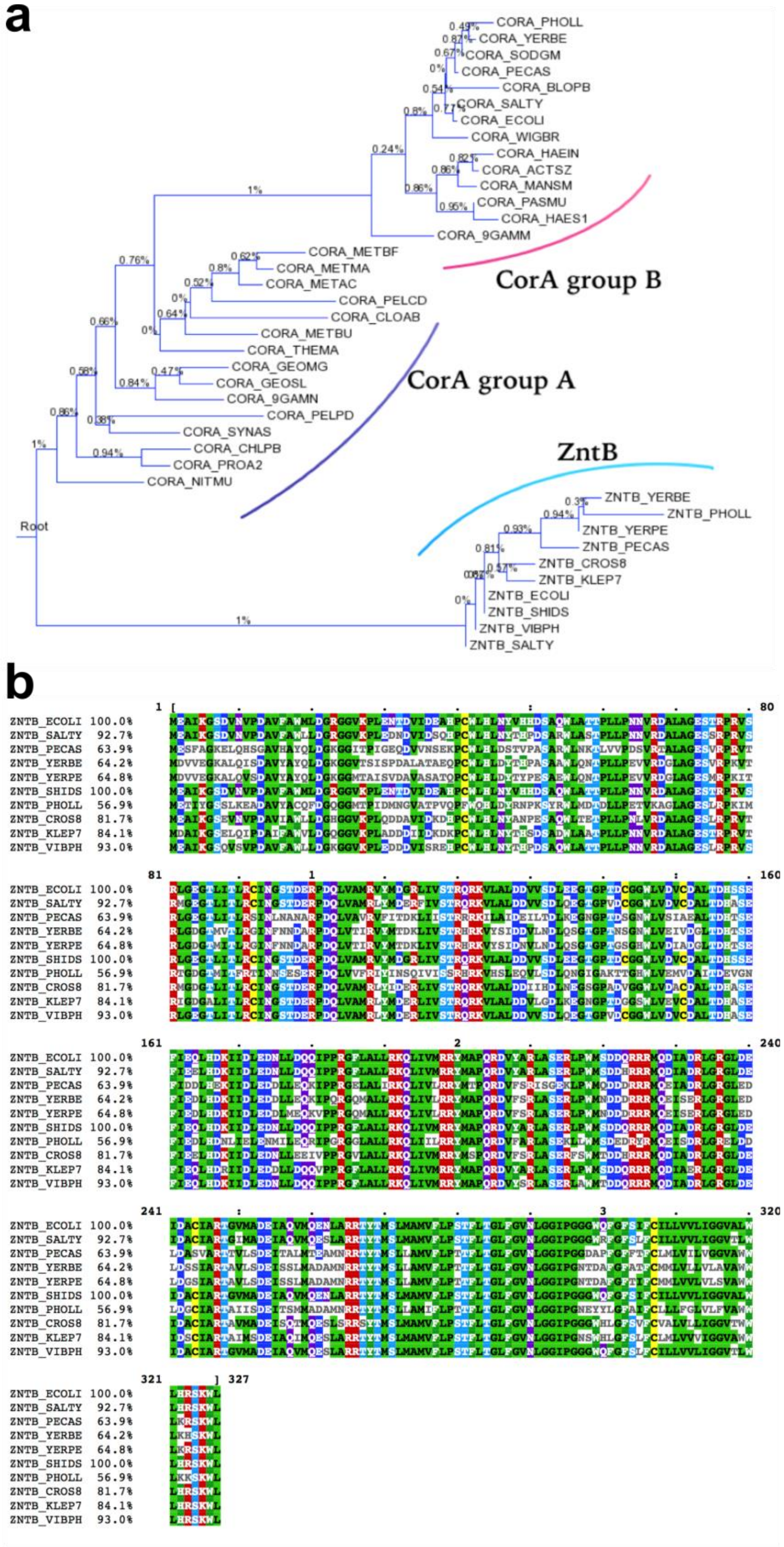


**Supplementary Figure 5 Phylogenetic tree and sequence analysis of ZntB proteins. (a)** ZntB proteins form a distinct clade of proteins, which are highly conserved as seen in (**b**) Sequence alignment of ZntB proteins, note the high sequence identity among EcZntB, StZntB anf VpZntB

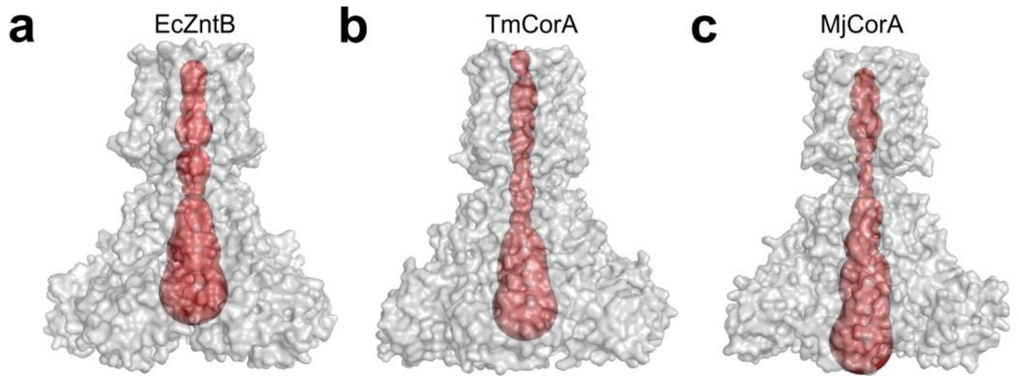


**Supplementary Figure 6 Calculated pore profiles of (a) EcZntB, (b) TmCorA, (c) MjCorA.** Proteins are shown as grey semi-transparent surface, pores in red. Note that the profile in ZntB is generally wider compared to CorAs.

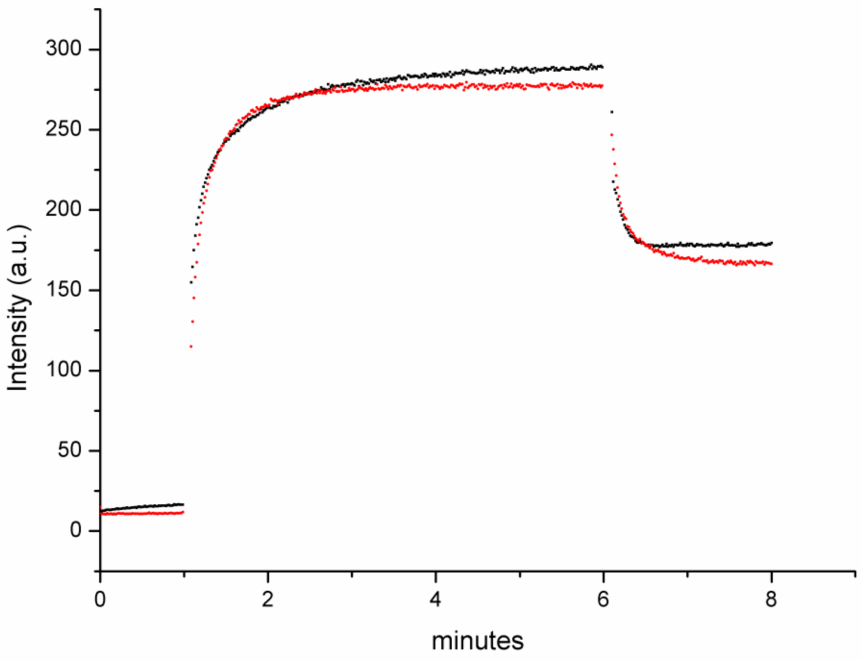


**Supplementary Figure 7 Fluorescent transport of $Zn^{2+}$ into proteoliposomes.** Sodium gradient (black line, $K^{+}_{inside}$ / $Na^{+}_{outside}$) does not stimulate uptake of $Zn^{2+}$ when compared to the standard condition (red line, $K^{+}_{inside}$ / $K^{+}_{outside}$). $Zn^{2+}$ and FCCP were added at 1 min and 6 min time points respectively.

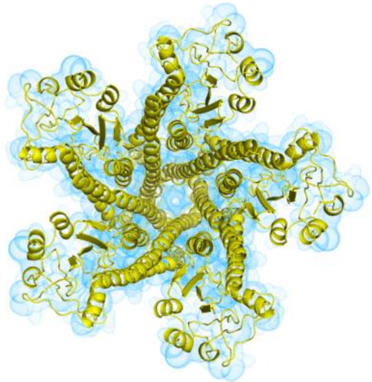

**Supplementary Figure 8 ZntB maintains its pentameric state upon additional EDTA treatment.** Cryo-EM reconstruction of ZntB with an extra 1mM EDTA added prior sample freezing. ZntB in yellow, Cryo-EM map in blue.
